# Editorial: Natural products: a microecological perspective for treating diabetes and its complications

**DOI:** 10.3389/fnut.2026.1896973

**Published:** 2026-07-22

**Authors:** Imran Khan

**Affiliations:** 1 Faculty of Medicine, Macau University of Science and Technology, Taipa, Macao SAR, China; 2 Department of Biotechnology, Faculty of Chemical and Life Sciences, Abdul Wali Khan University Mardan, Mardan, Khyber Pakhtunkhwa, Pakistan

**Keywords:** diabetes mellitus, drugs, herbal medicine, kidney—abnormalities, metabolism, microbiome, prebiotics, probiotics

## 1 Introduction

The rising incidence of Type 2 diabetes mellitus (T2DM) and its associated microvascular and macrovascular complications demand a change in treatment approaches (1). T2DM is a systemic disorder involving chronic inflammation, metabolic inflexibility, a disrupted intestinal barrier, and microbial dysbiosis. These modulations carry grave consequences in the form of tissue injuries across multiple organs and can cause serious health complications that range from nephropathy to osteoporosis (1, 2).

**Figure 1:**
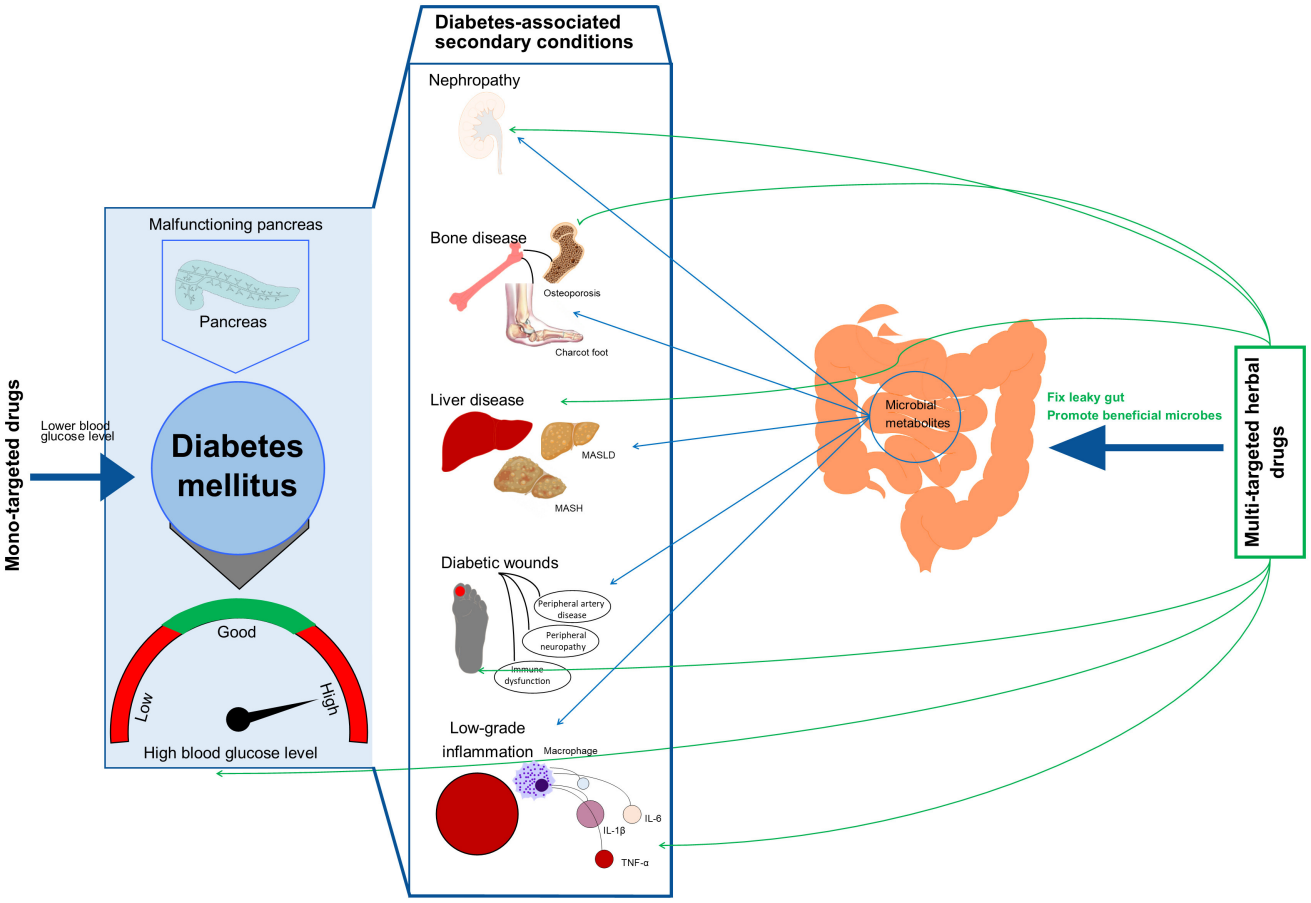
Graphical illustration of the mono-targeted, single-drug approach vs. the multi-targeted, herbal drug approach in the management of diabetes mellitus and its associated comorbidities. Mono-targeted drugs lower systemic blood glucose levels by either enhancing insulin secretion or improving peripheral insulin sensitivity. Multi-target drugs manage diabetes at multiple levels, ranging from gut microbiome improvement to lowering inflammation. Beyond primary glycemic control, multi-targeted herbal drugs also improve secondary conditions associated with diabetes. Microsoft Publisher was used to generate this image. No generative AI was used for the conceptualization or drafting of this figure.

It is believed that T2DM management could be improved through precision nutrition and herbal formulations, which could shift the paradigm from a single-pathway, single-organ understanding to a multi-target, integrated biology approach (Figure 1). This paradigm shift in T2DM treatment and management does not require the replacement of conventional therapies, but rather their expansion to include natural products as multi-target interventions, which may be especially well-suited to addressing this complexity and improving patients' health holistically (3) (Figure 1). To this end, Yang et al. reviewed the relevant literature and proposed that T2DM and associated Polyendocrine Metabolic Ovarian Syndrome (PMOS) could be managed by targeting the gut-ovary-metabolism axis through the combination of probiotics and metformin. We summarize the main findings of the articles collected in this Research Topic and discuss the growing enthusiasm for multi-target treatment strategies for T2DM treatment and management.

## 2 The gut microbiome: a metabolic nexus

It is well-known that the gut microbiome (GM) promotes metabolism by breaking down resistant dietary fibers into short-chain fatty acids (SCFAs), which play a key role in regulating lipid storage and glucose levels (4). The human intestinal barrier is highly selective and comprises a single layer of epithelial cells and apical junctional complexes, such as claudins, adherens junctions, occludin, and zonula occludens-1 (5). This equilibrium is disturbed during GM dysbiosis, a state in which microbial diversity is lost, obligate anaerobes decrease in the large intestine, and pathobionts (often facultative anaerobic *Enterobacteriaceae*) propagate in the bowel (6).

GM dysbiosis is frequently associated with metabolic disorders, such as obesity, T2DM, and hormonal irregularities. New findings indicate that GM dysbiosis is not just a consequence of T2DM but a driver of insulin resistance (IR) and systemic inflammation (7). Advances in computational models are helping distinguish individuals with IR from patients with T2DM based on GM cluster signatures. For instance, XGBoost identified *Bacteroides* and *Faecalibacterium* as key discriminators of IR severity (He et al.).

One of the widely accepted pathways through which GM dysbiosis can cause systemic inflammation is the “leaky gut” phenomenon, in which intestinal barrier damage leads to increased translocation of endotoxins and uremic toxins (e.g., indoxyl sulfate), which can initiate and exacerbate systemic inflammation and fuel polycystic ovary syndrome and diabetic kidney disease (7).

## 3 Healing leaky gut and fixing dysbiosis through prebiotics

Non-digestible carbohydrates (such as β-inulin or resistant starch) can restore depleted beneficial commensal strains. A clinical trial (the TarGut-CKD study) has performed the restoration of GM dysbiosis through prebiotics. Importantly, these prebiotics promote the growth of SCFA producers (such as *Faecalibacterium* and *Roseburia*), which help heal leaky gut by upregulating tight junction proteins such as Zonula Occludens-1 (ZO-1) (8).

Among the articles collected here, (Liu et al.) identified herbal monomers that regulate PPARγ/LXRα signaling and exert renoprotective functions by regulating lipid homeostasis and oxidative stress. Another study, by Dai et al., found that the Yiqi Huoxue recipe, derived from traditional Chinese medicine (TCM), has protective abilities in DKD through autophagy by affecting mitochondria-associated endoplasmic reticulum membranes and calcium homeostasis. In addition, several other studies have reported the beneficial effects of natural compounds and TCM formulas (such as Lycium, icariin, jiaogulan, barbarum polysaccharide, *Dendrobium officinale* polysaccharides, *Gastrodia elata* water extract, and cumin infusion) in regulating glucose and lipids, reinstating GM diversity and composition, and lowering inflammation, eventually benefiting multiple organ systems [Wan et al.; Jia et al.; (9–11)].

The beneficial role of TCM in improving GM composition and alleviating associated disease was well-explained by Yin et al.. Their review proposed that PCOS-induced IR and hyperandrogenism could be treated with TCM by restoring microbial balance, reducing low-grade inflammation, and improving IR.

## 4 TCM: a rich source of prebiotics

The TCM model is based on the holistic improvement of the body and aligns well with systems biology, providing an efficient alternative treatment and management strategy for complex diseases, such as metabolic syndromes, cardiovascular diseases, and neurodegenerative disorders (12). The diverse constituents of the TCM formulas target a wide range of drug receptors. In addition, TCM is a rich source of prebiotics (e.g., non-digestible carbohydrates, oligosaccharides, and polyphenols), which promote the growth of beneficial microbes (e.g., *Bifidobacterium* and *Lactobacillus*) in the colon. Consequently, they confer beneficial effects on the host (13). Furthermore, the GM serves as a metabolic mediator and biotransforms hydrophobic TCM components into smaller, more bioavailable, and biologically active metabolites. The relationship between TCM and GM is bidirectional: TCM improves gut health by remodeling GM, and beneficial bacteria improve TCM bioavailability and efficacy.

TCM could serve as an effective alternative strategy for the management of diabetes mellitus and its secondary complications (e.g., DKD and chronic wounds). TCM-derived prebiotics could address the root cause of diabetic complications through the gut-pancreas axis. According to He et al., there is compelling evidence that certain GM clusters are associated with IR-related clinical measures. *Dendrobium officinale* is another TCM herb known for managing T2DM. Wan et al. summarized the available literature and proposed that *Dendrobium officinale* polysaccharides, the major bioactive constituent of this herb, should be developed as a prebiotic for remodeling GM, which could eventually improve intestinal permeability and ameliorate T2DM symptoms.

### 4.1 TCM-derived prebiotics and their role in glucose and lipid metabolism

T2DM and metabolic dysfunction-associated steatohepatitis (MASH) are tightly linked chronic metabolic diseases that exacerbate each other and create a dangerous cycle of lipid and glucose dysregulation (14, 15). During diabetes mellitus, increased hepatic glucose causes lipid accumulation, contributing to the accelerated progression of metabolic dysfunction-associated steatohepatitis toward liver fibrosis and steatohepatitis (16). In a comprehensive review, Li et al. established a firm causal association between visceral adipose tissue accumulation and DKD, which is governed through a multichannel pathophysiological network.

The gut microbiota plays a key role in the progression of T2DM complicated by MASLD. Therefore, modulation of the gut microbiota has emerged as a key strategy for managing MASLD in patients with T2DM (17). Several studies have reported that the Zhaqu Compound (composed of eight TCM ingredients) alleviates MASLD and T2DM by remodeling GM and their metabolites, consequently improving hepatic glucose and lipid metabolism (18–20). Liu et al. found that the Zhaqu compound serves as a prebiotic for key genera such as *Bacteroides, Mucispirillum*, and *Lactobacillus*, and that it improves hepatocyte metabolic function through PPARγ-related pathways by secreting metabolites (such as SCFAs) ). In addition, *Lycium barbarum* polysaccharides have been shown to improve the glycolipid profile and protect against glucotoxicity-induced organ injury. Yao, Xing et al. proposed that *Lycium barbarum* polysaccharide-based supplements should be developed and prescribed for the efficient management of T2DM and associated disease conditions. In addition, Huang et al. evaluated a *Sanghuangporus vaninii*-based formulation for its anti-T2DM effects through network analysis and observed that apigenin and tangeritin were the most potent constituents of the formula, associated with decreased levels of M3G (a hepatic metabolite).

### 4.2 TCM-derived prebiotics and their role in diabetic nephropathy

The gut–kidney axis is a relatively new framework through which dysbiosis is implicated in the onset and progression of diabetic kidney disease (DKD) and chronic kidney disease (CKD). The interaction between the GM and the kidneys is bidirectional—exacerbated uremia in CKD and chronic hyperglycemia in DKD can remodel GM diversity and composition. Similarly, dysbiosis-induced decreases in anti-inflammatory metabolites (e.g., butyrate) and increases in endotoxins (LPS) could cause renal decline (21).

Diabetic nephropathy, also known as diabetic kidney disease (DKD) is a serious, progressive microvascular complication of diabetes mellitus. While conventional medications fail to provide therapy for DKD, TCM progresses through a systemic approach to mitigate intraglomerular hypertension. The Yiqi Huoxue Recipe is considered one such approach that could efficiently regulate DKD. Dai et al. found that the Yiqi Huoxue recipe protects the kidneys by targeting the mitochondria-associated endoplasmic reticulum membrane complex (VAPB–PTPIP51), which triggers autophagy, a key cellular cleanup process that safeguards podocytes from hyperglycemic damage. Yao, Xing et al. explored the underlying mechanisms of the Shenqi compound in NS-1 cells and reported that its protective effect operates through the NOD1/RIP2 signaling pathway and favors the maturation of insulin secretory vesicles. Sun et al. proposed that the gut microbiota-gut-kidney axis holds profound importance in DKD management and should be studied through precision nutrition. Through a comprehensive bibliometric analysis, Cui et al. determined that herbal interventions for DKD are growing rapidly and emphasized the need for deeper mechanistic research to strengthen confidence in herbal medications as evidence-based therapies. Wu C. et al. have explained that TCM monomers have considerable potential for managing DKD by regulating cholesterol efflux.

### 4.3 TCM-derived prebiotics and their role in healing diabetic wounds

Wu J. et al. explained the concept of the metabolism–senescence axis, through which diabetic wounds are sustained by inflammation and impaired processes driven by hyperglycemia and IR. During diabetic wound healing, mitochondria are dysfunctional, oxidative stress and glycation are higher, and senescent cells are aggregated. The metabolism–senescence axis well explains why multifunctional TCM is an emerging approach for treating diabetic wounds compared to agents that target a single node (Wu J. et al.).

### 4.4 TCM-derived prebiotics and their role in other conditions

Through a systematic review, Yang et al. summarized that T2DM and Polyendocrine Metabolic Ovarian Syndrome (PMOS) are linked through GM dysbiosis and IR. The authors proposed that polycystic ovary syndrome could be managed by targeting the gut-ovary-metabolism axis through the synergistic effects of probiotics and metformin. In another study, Yin et al. suggested that TCM has a strong foundation for alleviating GM dysbiosis, reducing inflammation, and improving IR. The authors listed TCM formulas that strengthen the intestinal barrier by promoting the growth of SCFA-producing bacteria, consequently lowering systemic and ovarian inflammation. Zhang et al. highlighted the importance of TCM in the treatment of another condition, diabetic gastroparesis, which commonly occurs with diabetic autonomic neuropathy and is characterized by abnormal and delayed gastrointestinal movement. Moreover, Jia et al. explored the potential of *Gastrodia elata* Blume (*G. elata*), an edible medicinal plant, for its therapeutic abilities against osteoporosis in streptozotocin–induced diabetic rats. Apart from TCM, Aslam et al. developed a carbohydrate-controlled diet combined with C*uminum cyminum*, a member of the Apiaceous family, which can provide support for glycemic control and lipid metabolism.

## 5 Conclusion

In conclusion, the articles in this Research Topic underscore a paradigm shift in the treatment of T2DM therapy, moving from pancreas- and glucose-centered management to a new holistic, multi-organ approach that harnesses the complexity of natural products through network pharmacology and targets the GM–organ axis system biology. The published articles in this Research Topic illustrate that herbal products—ranging from singular traditional monomers to complex herbal formulations—engage in bidirectional crosstalk with the host's internal microenvironment. Herbal products have considerable potential beyond simple glucose management, including tissue repair and treatment of chronic secondary conditions of T2DM. Since this research is currently in the preclinical phase, the translation of the findings to a large scale is the next critical step in validating them. Future studies should be conducted to overcome the limitations of bioavailability and efficacy, and the development of synbiotics could be one such innovative targeted system.
